# Platelet-Rich Plasma (PRP) in Dermatology: Cellular and Molecular Mechanisms of Action

**DOI:** 10.3390/biomedicines12010007

**Published:** 2023-12-19

**Authors:** Denisa Vladulescu, Lucian G. Scurtu, Anca Angela Simionescu, Francesca Scurtu, Marco I. Popescu, Olga Simionescu

**Affiliations:** 1 Faculty of Medicine, Carol Davila University of Medicine and Pharmacy, 050474 Bucharest, Romania; 2 Department of Dermatology I, Colentina Hospital, 020125 Bucharest, Romania; 3 Department of Obstetrics and Gynecology, Filantropia Clinical Hospital, 011132 Bucharest, Romania; 4 Faculty of Medicine, “Titu Maiorescu” University, 040441 Bucharest, Romania

**Keywords:** platelet-rich plasma, growth factors, alopecia, wound healing, melasma, vitiligo, scars, lichen sclerosus

## Abstract

Platelet-rich plasma (PRP) therapy has gained attention in the scientific field due to its potential regenerative effects and great benefit–risk ratio. This review extensively explores the most studied mechanisms of this therapy according to the etiopathogenesis of skin diseases: cellular proliferation, matrix formation, regulation of inflammation, angiogenesis, collagen synthesis, and the remodeling of new tissue. Moreover, it draws on newly reported and lesser-known effects of PRP: its anti-apoptotic effects, immunological suppression, decrease in melanin synthesis, anti-microbial effects, overexpression of miR-155, antioxidant effects, and their involved pathways. This work aims to provide a complete update for understanding PRP’s benefits and clinical relevance in wound healing, alopecia, pigmentary disorders, scars, rejuvenation, lichen sclerosus, and other inflammatory dermatoses, based on the current evidence. Furthermore, recent reports with novel indications for PRP therapy are highlighted, and new potential pathways correlated with the pathogenesis of skin diseases are explored.

## 1. Introduction

**Figure 1:**
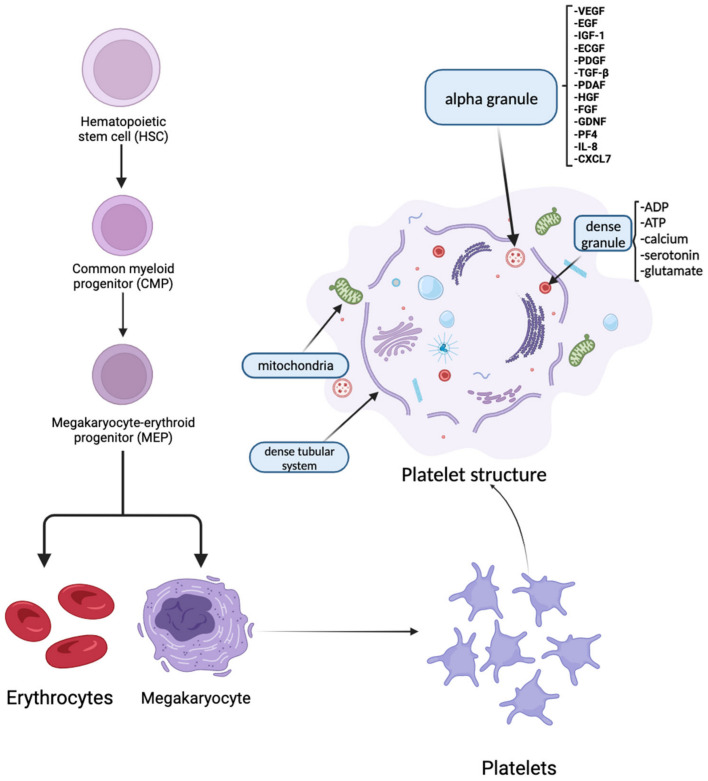
Platelet development and structure. The hematopoietic stem cell is a multipotent stem cell that represents the progenitor for blood cells and immune system cells. Common myeloid progenitors are derived from hematopoietic stem cells and give rise to megakaryocyte–erythroid progenitors. Both megakaryocytes and erythrocytes are differentiated from megakaryocyte–erythroid progenitors. Platelets are cell fragments developed through the cytoplasmic fragmentation of megakaryocytes. Alpha and dense granules from platelets have an important role in platelet-rich plasma therapy. VEGF = vascular endothelial growth factor; ECGF = endothelial cell growth factor; IGF-1 = insulin-like growth factor-1; PDGF = platelet-derived growth factor; TGF-β = transforming growth factor-β; EGF = epidermal growth factor; PDAF = platelet-derived angiogenesis factor; HGF = hepatocyte growth factor; FGF = fibroblast growth factor; GDNF = glial cell line-derived neurotrophic factor; PF4 = platelet factor 4; IL-8 = interleukin 8; CXCL7 = chemokine (C-X-C motif) ligand 7; ADP = adenosine diphosphate; ATP = adenosine triphosphate.

Platelets are small blood cell fragments derived from megakaryocytes. They have a fundamental role in hemostasis and carry two types of storage granules: alpha-granules and dense granules (Figure 1) [1,2]. Alpha-granules are the most relevant to platelet-rich plasma (PRP) therapy due to their high number of growth factors: vascular endothelial growth factor (VEGF), endothelial cell growth factor (ECGF), insulin-like growth factor-1 (IGF-1), platelet-derived growth factor (PDGF), transforming growth factor-β (TGF-β), epidermal growth factor (EGF), platelet-derived angiogenesis factor (PDAF), hepatocyte growth factor (HGF), fibroblast growth factor (FGF), glial cell line-derived neurotrophic factor (GDNF), platelet factor 4 (PF4), interleukin 8 (IL-8), and β-thromboglobulin (or CXCL7). Dense granules are the second-most abundant granules in platelets after alpha-granules. Their storage of ADP, ATP, calcium, serotonin, and glutamate, which is released after PRP treatment, plays an important role in the beneficial effects of this therapy [2,3,4].

Platelet-rich plasma (PRP) therapy is a method that uses a centrifugated blood fraction containing a high concentration of platelets in a small volume of plasma. The optimal platelet concentration recommended for high-quality PRP treatment in skin diseases is 1–1.5 million platelets/μL [5]. The secretion of growth factors from alpha-granules begins 10 min after injection with PRP, and at least 95% of them are released within one hour. For seven days, the platelets continue to produce and release additional growth factors. Cells within the injection site express growth factor receptors, whose activation induces positive results such as cellular proliferation, matrix formation, regulation of inflammation, angiogenesis, osteoid production, collagen synthesis, and the remodeling of novel tissues [6,7]. PRP therapy has a good safety profile, with few absolute contraindications: critical thrombocytopenia; platelet dysfunction; hemodynamic instability; and sepsis and local infection at the site of PRP administration. Relative contraindications include nonsteroidal anti-inflammatory drug use within 48 h before treatment, glucocorticoid injection use within 2 weeks before treatment, recent illness or fever, malignancies, anemia (with a hemoglobin level of less than 10 g per deciliter), mild thrombocytopenia, and tobacco use [8].

A big interest has developed for this therapy in the medical field over the last few years due to its satisfying benefit–risk ratio, with approximately 8000 papers being released since the earliest studies on this topic—of which more than 6000 were in the last 10 years, according to PubMed. PRP was first described in hematology in the 1970’s and was used for the treatment of patients with thrombocytopenia. Later on, in the early 1990’s, promising results were noticed when using PRP as a monotherapy or combined therapy for various medical conditions [9]. In dentistry, PRP has brought additional benefits in oral and maxillofacial surgery, and superior outcomes were noticed in regenerative endodontics and periodontics [10]. In plastic and reconstructive surgery, PRP has been described as valuable, especially for wound healing, fat, and bone graft regeneration [11]. A big expansion in the use of PRP, with encouraging results, has been displayed in orthopedics for bone fracture healing, injuries of ligaments, muscles, and tendons, articular cartilage lesions, and peripheral nerve injuries [12]. Asherman’s syndrome, premature ovarian failure (POF), wound healing, rejuvenation, and urinary incontinence represent the main PRP applications in gynecology and is a hot topic today [13].

In aesthetics and medical dermatology, PRP therapy continues to show more and more promising results in wound healing, non-scarring and scarring alopecias, rejuvenation, scars, pigmentary disorders, inflammatory disorders, and mucosal and nail disorders [14,15,16].

Platelet-rich plasma is a non-specific term that comprises four different products: pure platelet-rich plasma (P-PRP) or leukocyte-poor platelet-rich plasma, which is the most used product in dermatology; leukocyte-platelet-rich plasma (L-PRP), mainly used in orthopedic disorders and wound healing; pure platelet-rich fibrin (P-PRF), used mostly in oral and maxillofacial surgeries; and leukocyte and platelet-rich fibrin (L-PRF), which is a useful tool for wound healing, periodontics, and sports medicine [17]. A study that measured leukocyte and erythrocyte concentrations in healthy volunteers described 14.9 ± 4.5 (10^3^/mL) as the most common value for leukocytes in L-PRP and 0.2 ± 0.2 (10^3^/mL) as the most common value for leukocytes in P-PRP. Regarding erythrocyte concentration, L-PRP contains an approximately 0.7-fold blood concentration and P-PRP contains very few erythrocytes [18].

The benefits of high leukocyte-concentration platelet-rich plasma therapy are controversial. The risk of catabolic effects through proteinase and proinflammatory cytokine release has been described, but also the benefits of providing better antimicrobial activity and better clinical outcomes [19].

An improvement in the understanding of the PRP mechanism of action in certain diseases may enhance the confidence of both physicians and patients. This may lead to more evidence of the therapy’s results and could extend its applicability to other disorders.

## 2. Wound Healing

PRP therapy has been successfully used for the treatment of different wounds. In dermatology, beneficial results have been recorded in various ulcers—mostly pressure, venous leg, and diabetic ulcers, and a few cases of pyoderma gangrenosum [14,20]. The main pathways involved are revascularization via proangiogenic properties, regeneration, and the replacement of damaged tissue. Furthermore, an antimicrobial effect has been suggested to be provided by PRP [21].

Wound healing involves a complex process that occurs through dynamic interactions between different types of cells and other molecules such as growth factors [22]. VEGF and PDGF are the principal growth factors involved in angiogenesis, aiding in the revascularization of damaged tissues [23]. Moreover, VEGF stimulates the release of matrix metalloproteinases (MMPs) and PDGF promotes the production of IGF-1 and TGF-β [24,25]. They also possess chemotactic effects on fibroblasts, monocytes, and neutrophils and stimulate collagen synthesis. HGF has an indirect role in angiogenesis and regeneration by stimulating VEGF activation [26]. Both TGF-β and FGF stimulate regeneration by providing a renewal of tissue through the activation of fibroblasts [27].

The FGF family encompasses 23 members that drive the expression of other growth factors in cells. Among these, FGF-2, FGF-7, FGF-10, and FGF-12 are more specific to skin. FGF-2, FGF-10, and FGF-12 modulate fibroblast cell migration and can activate the c-Jun N-terminal kinase (JNK) pathway [28]. TGF-β1 stimulates the production of fibronectin and collagen, and TGF-β3 prevents excessive scar formation [26,29]. EGF contributes to the replacement of damaged tissue by its proliferative influence on all epithelial cell types [29,30]. IGF-1 has an important role in the inflammatory and proliferative phase of skin wounds [24]; it promotes the differentiation of new cells, stimulates extracellular matrix synthesis, and increases membrane glucose transport in cells [26,29].

**Figure 2:**
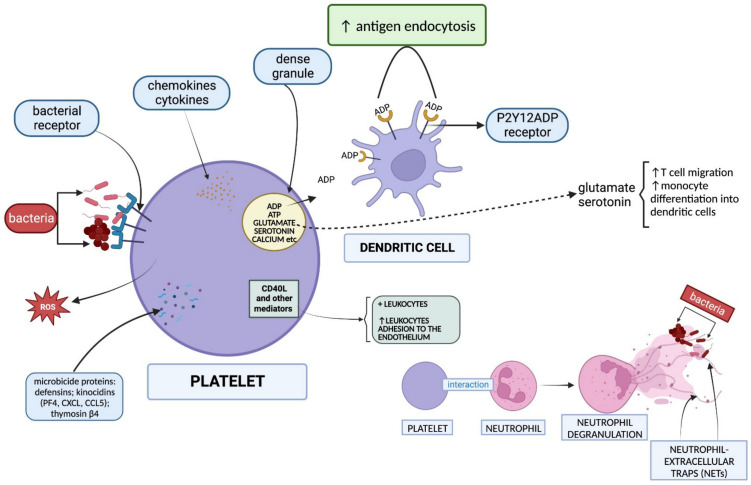
Mechanisms involved in the antimicrobial activity of platelets. Platelets provide direct antimicrobial effects by releasing cytoplasmic components (microbicide proteins, chemokines, and cytokines), generating reactive oxygen species and promoting bacterial receptor expression. Indirect antimicrobial activity is provided via interactions with leukocytes and dendritic cells. ADP = adenosine diphosphate; ATP = adenosine triphosphate; ROS = reactive oxygen species; PF4 = platelet factor 4; CXCL = chemokine (C-X-C motif) ligand; CCL5 = chemokine (C-C motif) ligand 5; CD40L = cluster of differentiation 40 ligand.

Infection is the most frequent complication of ulcers and it affects the healing of any kind of wound. PRP seems to generate antimicrobial activity in vitro and in vivo (Figure 2) [31]. An antimicrobial effect of some chemokines and cytokines released by platelets has been reported, which can recruit and activate immune cells. PRP also contains microbicide proteins: kinocidins (PF4, CXCL, and CCL5), defensins, and thymosin β4 [21]. Additionally, platelets express potential bacterial receptors that can internalize bacteria. They also generate reactive oxygen species that participate in antibody-dependent cellular cytotoxicity [32].

Thrombocytes recruit and modulate leukocytes’ behavior mainly through their soluble mediators, e.g., CD40L. These mediators can directly activate leukocytes and can support their adhesion to the endothelium by activating endothelial cells and inducing the expression of adhesion molecules [33]. The interaction between platelets and neutrophils leads to neutrophil degranulation, with the formation of neutrophil-extracellular traps (NETs). NETs are networks of fibers composed of intracellular neutrophil components with the ability to trap and kill bacteria [34]. ADP, a constituent of dense granules, can bind to dendritic cells (DCs) through the P2Y12ADP receptor and increase antigen endocytosis. Other constituents of dense granules such as glutamate and serotonin provide immunomodulatory effects by inducing T-cell migration and increasing monocyte differentiation into DCs. DCs are also important for connecting innate to adaptive immune systems [34,35,36]. Through similar mechanisms, reports suggest that PRP could be a helpful tool for burn management, although the evidence is weak [25,37]. Moreover, PRP can reduce burn pain in rat model experiments [38].

## 3. PRP in the Treatment of Hair Disorders: Androgenetic Alopecia and Alopecia Areata

**Figure 3:**
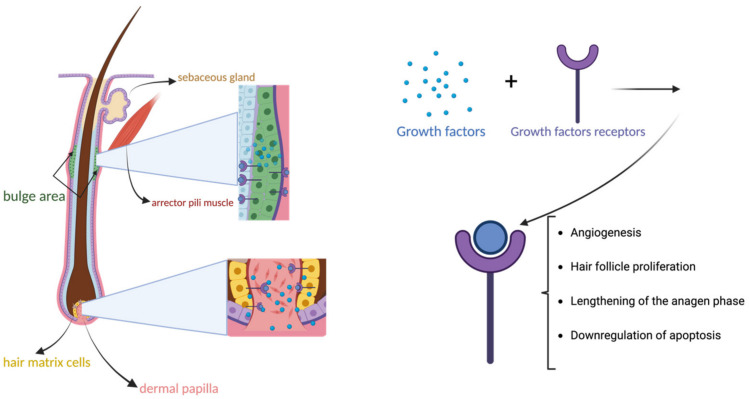
Structures involved in platelet-rich plasma therapy’s effects on hair follicles. The bulge area and the matrix area are the two main hair follicle sites containing hair follicle stem cells that express growth factor receptors. The bulge area is located between the attachment site of the arrector pili muscle and the opening of the sebaceous gland. The matrix area is situated around the dermal papilla at the bottom of hair follicles. By binding to their receptors on hair follicle stem cells, growth factors exert their beneficial effects on alopecias.

Non-scarring alopecias are among the most studied uses of PRP therapy. Many of the growth factors released by activated platelets seem to influence hair growth by promoting angiogenesis, hair follicle cell proliferation, and lengthening of the anagen phase. They exert these actions by binding to their receptors on stem cells from the bulge area and on germinative cells of mesenchymal origin from the matrix (Figure 3) [23]. Moreover, apoptosis is downregulated through PRP therapy via the activation of B-cell lymphoma 2 (BCL-2) and by stimulation of the ERK/protein kinase B (Akt) signal pathway [39].

VEGF and PDGF have proangiogenic properties, thereby increasing perifollicular vascularization. VEGF also stimulates microvascular permeability and PDGF up-regulates the genes associated with the induction and control of anagen [23,40]. IGF plays a significant role in hair growth by promoting cell proliferation; it extends the anagen phase and prevents hair follicles from entering the catagen phase. Lower EGF receptor signaling generates deficient hair follicle genesis in mice. EGF effects have also been studied in vitro, on human follicles, where it enhances elongation and sustains the growth of follicle cells [30,41,42]. Moreover, associated EGF and IGF therapy has a positive impact on the transition from the telogen to the anagen phase, stimulating hair growth in the angora rabbit [30]. A similar effect is suggested for FGF, which promotes hair growth by inducing the anagen phase and prolonging the mature anagen phase of the hair follicles of a mouse model [27]. Expression of GDNF and its receptor on hair follicle cells seems to stimulate hair proliferation and keep the hair in the anagen phase [4]. Studies on mice have revealed that subcutaneous injection of platelet-derived ECGF (PD-ECGF), PDGF-C, and chemokine (C-X-C motif) ligand 1 (CXCL 1) stimulates anagen induction in mice and the proliferation of dermal papilla cells. Furthermore, combination therapy between minoxidil and PD-ECGF, PDGF-C, and CXCL 1 is more effective in anagen induction than minoxidil alone [43]. PDGF is also produced by endothelial cells and keratinocytes and stimulates cell interactions that are required for hair canal development [16]. FGF-7 is a growth factor located in dermal papilla cells that prolongs the anagen phase. β-catenin is a protein that stimulates hair follicle development and whose levels are correlated with hair growth. PRP increases FGF-7 levels and β-catenin activity in hair follicles [16,44,45]. In vitro and in vivo studies have demonstrated that PRP activates the ERK and Akt pathway, thereby regulating cell growth via ERK signaling and prolonging cell survival via Akt anti-apoptotic effects. The prevention of hair follicle cell apoptosis is also achieved through the increasing of levels of BCL-2, induced by PRP therapy [16,45]. Additionally, an increase in a marker of cell proliferation (Ki-67) is displayed in androgenetic alopecia and alopecia areata after treatment, suggesting enhanced cell proliferation [16,46]. A study that evaluated results following three months of PRP therapy—three treatment sessions, one every 30 days—compared to a placebo for androgenetic alopecia, showed an increase in epidermal thickness, Ki-67 positive keratinocytes, the number of follicles, the number of hairs, and total hair density for the target area compared to the placebo [45].

**Figure 4:**
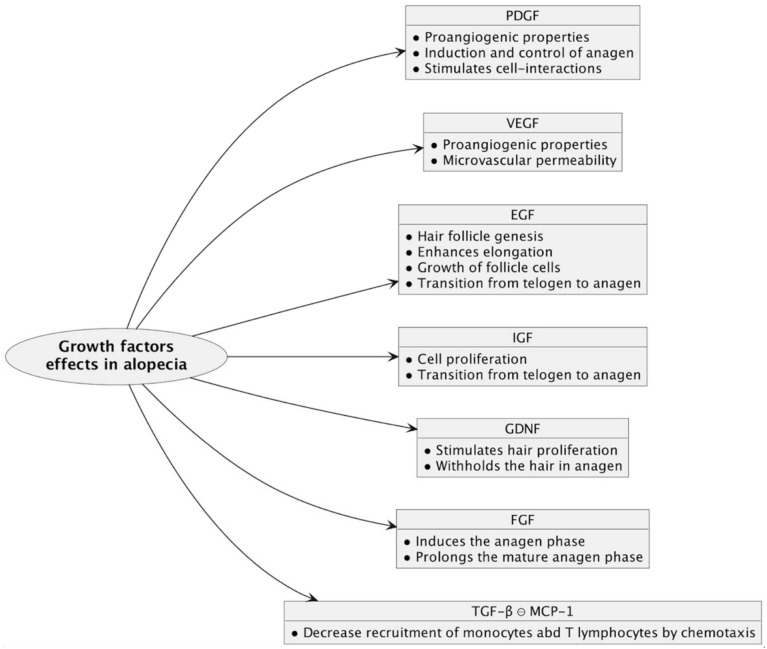
Growth factors and their mechanisms in alopecia. Angiogenesis, hair follicle cell proliferation, and prolongation of the anagen phase are the main effects of platelet-rich plasma therapy, responsible for restoring the normal hair growth cycle in non-scarring alopecias. These effects are achieved through the synergistic actions of multiple growth factors. PDGF = platelet-derived growth factor; VEGF = vascular endothelial growth factor; EGF = epidermal growth factor; IGF = insulin-like growth factor; GDNF = glial cell line-derived neurotrophic factor; FGF = fibroblast growth factor; TGF-β = transforming growth factor-β; MCP-1 = monocyte chemoattractant protein-1.

The immunological suppression of PRP mediated by TGF-β might be another mechanism providing beneficial results in patients with alopecia areata. TGF-β represents an inhibitor of MCP-1, a cytokine that recruits monocytes and T lymphocytes via chemotaxis. Moderate MCP-1 expression in hair follicles has been associated with alopecia areata [17,47]. All growth factors’ effects on alopecia are summarized in Figure 4.

## 4. Pigmentary Disorders

To date, the mechanisms of PRP therapy in melasma and vitiligo are not completely understood.

### 4.1. Melasma

**Figure 5:**
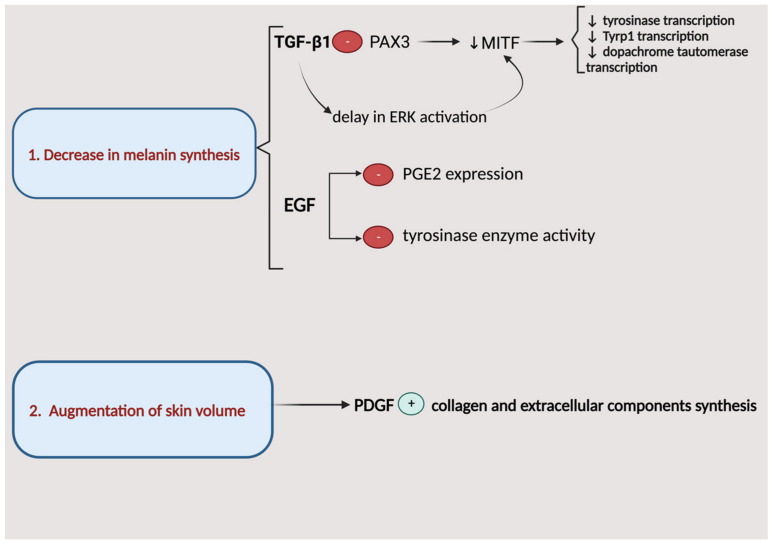
Action mechanisms of platelet-rich plasma therapy in melasma. Among the growth factors delivered through platelet-rich plasma therapy, TGF-β1, EGF, and PDGF are the main factors involved in the reversal of melasma pathogenesis. TGF-β1 = transforming growth factor-β1; PAX3 = paired box homeotic gene 3; MITF = microphthalmia-associated transcription factor; ERK = extracellular signal-regulated kinase; EGF = epidermal growth factor; PGE2 = prostaglandin-E2; PDGF = platelet-derived growth factor.

The two main reported mechanisms responsible for PRP’s effects in melasma are a decrease in melanin synthesis mediated by an isoform of TGF-β (TGF-β1) and EGF, and augmentation of skin volume through the stimulation of collagen and extracellular component synthesis, mainly by PDGF (Figure 5) [48]. TGF-β1 directly inhibits paired-box homeotic gene 3 (PAX3) expression, which down-regulates microphthalmia-associated transcription factor (MITF). MITF regulates the transcription of tyrosinase, tyrosinase-related protein 1 (Tyrp1), and dopachrome tautomerase. Moreover, TGF-β1 induces a delay in the activation of extracellular signal-regulated kinase (ERK) in an immortalized mouse melanocyte cell line, and ERK pathway activation has been found to inhibit tyrosinase gene transcription. Thus, TGF-β1 mediates an indirect inhibition of melanogenesis [49,50,51]. TGF-β1 also stimulates laminin, collagen IV, and tenascin, providing a healing effect for the skin’s basement membrane [52]. EGF suppresses prostaglandin-E2 (PGE2) expression and tyrosinase enzyme activity in laser-treated keratinocyte-conditioned culture media [53]. Since the levels of interleukin-17, leukocyte differentiation antigen-4, and cyclooxygenase-2 are higher in melasma-affected skin, PRP’s anti-inflammatory effects could also be involved in its beneficial results for this condition [54,55].

### 4.2. Vitiligo

**Figure 6:**
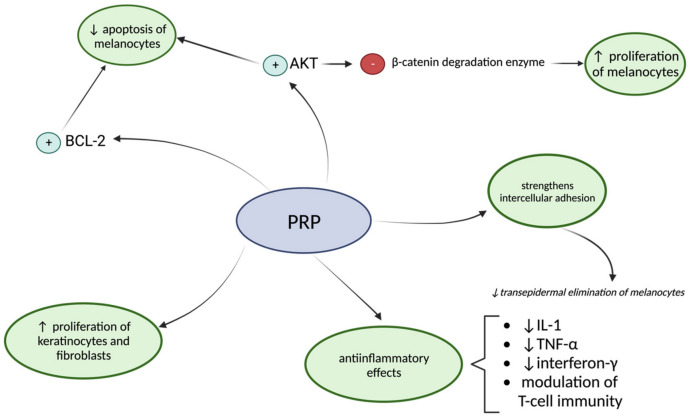
Beneficial effects of platelet-rich plasma for vitiligo. Melanocytes are the main cells involved in vitiligo pathogenesis. Through platelet-rich plasma therapy the preservation of melanocytes’ integrity and activity is accomplished. PRP = platelet-rich plasma; BCL-2 = B-cell lymphoma 2; AKT = protein kinase B; IL-1 = Interleukin-1; TNF-α = tumor necrosis factor-α.

PRP has been reported as a potential treatment for vitiligo by promoting melanocyte regeneration, inducing a strengthening of intercellular adhesion, and providing anti-inflammatory effects (Figure 6) [47]. The ability of the growth factors from PRP to stimulate Akt and BCL-2 prevents melanocyte apoptosis. Akt inhibits the enzyme which degrades β-catenin and therefore determines β-catenin inflation, which stimulates the proliferation of melanocytes [56]. It has been hypothesized that a deficiency of FGF and KGF could lead to a weak attachment between melanocytes and other skin cells, causing a transepidermal elimination of melanocytes [57]. PRP contains fibrin, fibronectin, and vitronectin, which contribute to the strengthening of intercellular adhesion. Moreover, growth factors also stimulate the proliferation of keratinocytes and fibroblasts and modulate the interaction between these cells and melanocytes [58,59]. The anti-inflammatory effects of the therapy are indicated by its capacity to down-regulate the release of interleukin-1 (IL-1), interferon-γ, and tumor necrosis factor-α—cytokines involved in the pathogenesis of vitiligo [60]. Additionally, TGF-β is involved in the modulation of T-cell immunity, thus providing supplementary anti-inflammatory effects [61].

## 5. Rejuvenation and Scars

In aesthetic medicine, PRP therapy has become an important tool through its positive effects on skin rejuvenation. Concurrently, or as a free-standing recommendation, it is often used to improve the appearance of acne scars [62]. Good results are also reported when using PRP for atrophic, keloidal, or hypertrophic scars of different etiologies [63].

In older, sun-damaged skin, a decrease in elastin is noticed, with disorganized, fragmented elastin fibers, reduced fibroblast activity, and a low capacity for synthesizing type I procollagen [64]. PRP therapy improves skin quality and reverses this natural aging process through the proliferation of fibroblasts and the up-regulation of MMP expression, with stimulation of collagen synthesis and remodeling of the extracellular matrix [47]. A noteworthy mechanism of PRP therapy in rejuvenation is the stimulation of intracellular antioxidant enzymes that counteract the oxidative stress secondary to UV exposure [65].

Fibroblasts are key factor cells in rejuvenation and scarring due to their intercellular interactions, mainly with keratinocytes and endothelial cells, and due to their ability to produce extracellular matrix proteins and cytokines [47,66]. PRP therapy influences fibroblast proliferation in a dose-dependent manner [25]. The activation of fibroblasts is achieved mainly through TGF-β and FGF [27]. TGF-β1 enhances the production of type I collagen and decreases collagen disintegration [67]. Increased MMP-1 and MMP-2 fibroblast expression determines the removal of fragmented fibrils and a proper remodeling of the extracellular matrix [68,69]. The trauma caused by injecting PRP also contributes to a positive outcome through the effects of the therapy on wound healing [70]. Similarly, the inadequate healing process of hypertrophic scars with insufficient degradation of the extracellular matrix, increased type III collagen, and decreased type I collagen is reversed [37,71].

Better skin quality and overall appearance of the skin are also achieved upon PRP reduction of hyperpigmentation through the above-described molecular mechanisms for melasma [48,72].

## 6. Lichen Sclerosus and Other Inflammatory Disorders

### 6.1. Lichen Sclerosus

The promising results of PRP therapy for lichen sclerosus represent a big achievement for the dermato-venereology field and regenerative medicine, even if most of the studies have been uncontrolled trials [73].

**Figure 7:**
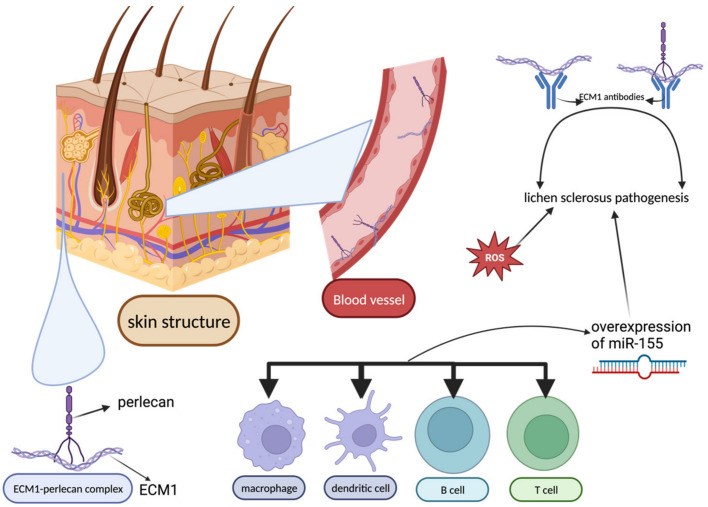
Skin structures and cells correlated with lichen sclerosus pathogenesis. The three most suggested mechanisms are circulating antibodies for extracellular matrix protein 1, oxidative stress, and overexpression of miR-155 in various cells. ECM1 = extracellular matrix protein 1; ROS = reactive oxygen species.

Lichen sclerosus (LS) is a chronic disease of unknown etiology characterized by inflammation, scarring, and atrophy, which most commonly affects the anogenital region [74]. Circulating antibodies for extracellular matrix protein 1 (ECM1) have been detected in most patients with LS. This explains the epidermal atrophy, as the extracellular matrix 1b isoform of the protein contributes to keratinocyte differentiation. Moreover, ECM1 binds perlecan, a proteoglycan that has an important role in skin remodeling. The expression of both ECM1 and perlecan in dermal blood vessels supports their role in angiogenesis and may explain the purpuric lesions seen in some patients with LS [75]. Nevertheless, it is not clear if the anti-ECM1 antibodies represent the root or the consequence of LS [76,77]. Other possible factors involved in LS pathogenesis are oxidative stress and overexpression of miR-155 (Figure 7). MiR-155 is a microRNA expressed in macrophages, dendritic cells, B cells, and T cells. miR-155 overexpression is associated with disruption of the suppressive function of T regulatory cells, further loss of self-tolerance, inflammation, and exaggerated collagen synthesis with successive sclerotic tissue formation [77,78].

Many of the released growth factors stimulate extracellular matrix remodeling, while TGF-β3 maintains a normal healing process and inhibits exaggerated scarring [26,29,79]. Angiogenesis and the promotion of cell proliferation are responsible for the reversal of skin atrophy following PRP [73,80].

Patients with LS are at risk of developing squamous cell carcinoma (SCC); a risk of up to 3.88% for women and up to 0.91% for men. PRP provides an antioxidant effect, thereby decreasing inflammation and preventing the inactivation of tumor suppressor genes such as p53 and CDKN2A. Hence, in addition to the above-mentioned clinical benefits, PRP may decrease the chances of SCC occurrence [65,81,82].

### 6.2. Psoriasis

PRP reduces the transactivating activity of nuclear factor kappa B (NF-κB), a protein transcription factor with a role in inflammatory processes, cellular proliferation, and apoptosis. Although the pathogenesis of psoriasis is still incompletely understood, NF-κB could act as a link for dysfunctional keratinocytes and immune cell interaction [83,84]. PRP therapy can reduce the activity of NF-κB. Additionally, a decreased release of proinflammatory cytokines has been noticed using PRP therapy. Hence, PRP therapy could induce anti-inflammatory activity via multiple pathways [60,85].

### 6.3. Behçet Disease

Patients with Behçet disease usually have lower levels of regulatory T cells. TGF-β and PF4/CXCL4 may modulate T-cell immunity and may contribute to the differentiation of regulatory T cells. A pilot study including six patients with less-serious Behçet disease on low doses of prednisone, for whom nine PRP therapy sessions were performed through a periumbilical subcutaneous application—the first six with a 15-day interval and the other three with a 30-day interval—revealed an increase in the frequency of Treg cells [86]. However, regarding Behçet disease or other inflammatory disorders, it is not very safe to assume that PRP from peripheral blood has immunoregulatory properties. Additionally, an experimental animal model study found low sera titers of IL-6 and IL-1 after PRP therapy, supporting its anti-inflammatory benefits [83].

### 6.4. Morphea

Favorable cosmetic outcomes with minimal side-effects in patients with localized morphea who underwent PRP have been described. The mechanisms of action involved are still to be elucidated but the stimulation of new collagen synthesis, the improvement of extracellular matrix remodeling, and anti-inflammatory effects were proposed as potential pathways [87,88].

### 6.5. Inflammatory Nail Disorders

Several case reports have shown good results after intramatriceal injections of PRP for nail lichen striatus, idiopathic trachyonychia, and lichen planus-associated nail dystrophy. The proposed mechanisms of action are anti-inflammatory effects and the beneficial effects of growth factors from alpha-granules on proliferation and keratinization [89,90].

### 6.6. Oral Lichen Planus

Two randomized clinical studies, each of them conducted on 20 patients, found intralesional PRP to be efficient for oral lichen planus, similar to triamcinolone acetonide [91,92].

A high ratio between reactive oxygen species and intracellular antioxidants was related to inflammation in oral lichen planus. An in vitro study showed that VEGF within alpha-granules activates nuclear factor (derived-erythrocyte) type 2 (Nrf2), which may prevent oxidative damage [93,94]. Additionally, the granules release anti-inflammatory cytokines such as IL-1 receptor antagonist (IL-1ra), soluble necrosis factor receptor (sTNF-R) I and II, IL-4, IL-10, IL-13, thereby providing anti-inflammatory effects in oral lichen planus [94,95,96].

## 7. Discussion and Future Directions

PRP therapy is a regenerative therapy which continues to be explored. Recently, platelet-rich plasma produced from cord blood units has been used for cord blood platelet gel formation; this represents a step forward in pediatric dermatology for epidermolysis bullosa’s treatment. Good results have also been accomplished for spinal reflex recovery in animal models, wound healing, and reconstruction of deep surgical sites after cardiothoracic surgery. Considering the huge amount of cord blood discarded from most cord blood banks (more than 80%) due to a low stem cell count, and the similar quantity of platelets with no distinction regarding their function contained in both cord blood units and adult peripheral blood samples, cord blood platelet gel production should be considered when possible [97,98,99]. Moreover, superior therapeutic effects for cord blood PRP over adult peripheral blood PRP have been suggested due to a more anti-inflammatory molecule profile for cord blood PRP than adult peripheral blood PRP [100].

**Figure 8:**
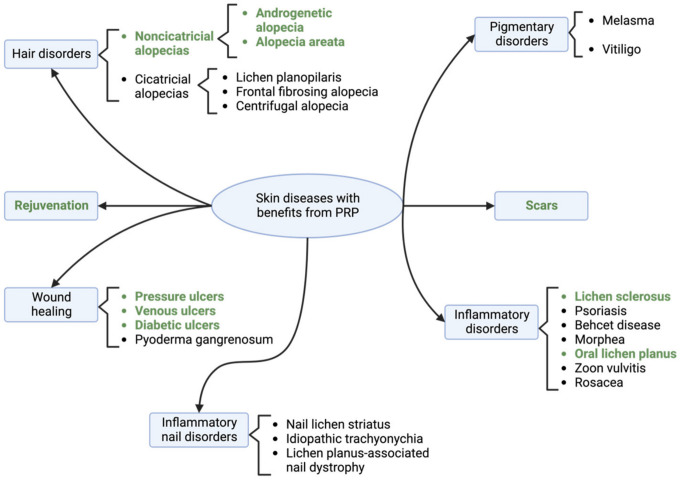
Skin diseases with reported favorable outcomes after platelet-rich plasma therapy. The most studied ones are highlighted. PRP = platelet-rich plasma.

Many skin disorders have a chronic evolution and require long-term therapy, which may be challenging due to adverse effects, low therapeutic responses, and relapses [101]. PRP may represent a long-lasting option for many of these pathologies, with a great benefit–risk ratio (Figure 8) [47,102,103].

A limitation of our understanding in terms of the action mechanisms of PRP therapy in dermatology is the unclear etiopathogenesis of many skin disorders [104]. For gaining improvements in clinical outcomes through PRP therapy, some pathways are extensively reported on, but there are also the less-studied ones, which might generate good results for further research. The effect of PRP on cell proliferation, lengthening of the anagen phase of the hair follicle, and anti-apoptotic activity provide benefits in non-scarring alopecia [16,105]. On top of this, there are a few reports that describe improvements in hair density, perifollicular erythema, and scaling for scarring alopecias, such as lichen planopilaris (LPP), frontal fibrosing alopecia (FFA), and centrifugal alopecia [106].

TGF-β1 and EGF’s synergistic effects in decreasing melanin synthesis are relevant not only for melasma but also for enhancing skin rejuvenation and improving post-inflammatory hyperpigmentation [48,72]. For acne scars, wound healing, hypertrophic scars, and lichen sclerosus, the stimulation of PRP on fibroblasts with the regulation of extracellular matrix remodeling is very promising [79,104].

Antimicrobial peptides such as defensins have turned out to be host defenders with bactericidal effects that can activate the immune system [35]. Defensins and other microbicide proteins are delivered through PRP therapy [21]. PRP antimicrobial activity is of major importance in wound healing, as infections often complicate ulcers. This also protects local infections at the injection site and shows benefits in rosacea associated with Demodex spp. infection [21,32,107]. Better outcomes and fewer complications have been noted for PRP therapy compared to conventional treatments (retinoids or lasers) for rosacea patients. Yet incompletely understood, rosacea etiopathogenesis involves dysregulation of innate and adaptive immunity, ultraviolet exposure, and inflammatory mechanisms that could be reversed by anti-inflammatory effects, immunological suppression, and the antioxidant effects provided by PRP therapy [107].

Regarding the atrophy that characterizes LS and the negative influence of LS on quality of life and sexuality, the angiogenesis and cell proliferation provided by PRP are of major importance [108,109]. Zoon vulvitis, a pathology that also affects the genital area and has a similar clinical appearance and evolution to LS, can be treated successfully with PRP, although the evidence for this is weak [110,111,112]. Considering the clinical similarity and evolution between LS and genital lichen planus, as well as the effectiveness reported after using PRP therapy for oral lichen planus and nail lichen planus, further investigation of its potential for genital lichen planus is also warranted [90,91,92,113].

Future directions regarding PRP therapy involve the improvement of scientific proof by conducting more randomized clinical trials. Additionally, observing the mechanisms of action already reported and correlating them to the pathogenesis of other dermatological diseases could help in extending the scope of PRP therapy studies and their recommendations.

## 8. Conclusions

PRP represents an expanding therapy that provides promising results for patients resistant to conventional treatments, as well as others. There is strong evidence for PRP benefits in non-scarring alopecias, wound healing, scars, and skin rejuvenation. PRP mechanisms for pigmentary diseases with antagonistic features such as melasma and vitiligo remain controversial. Given the significant clinical improvements in lichen sclerosus, this therapy was applied for Zoon vulvitis with promising results. Still, there is room for research and conceivably for extending recommendations to other diseases with similar evolutions and clinical features. Studies regarding PRP’s usefulness in inflammatory nail disorders, autoimmune diseases, rosacea, and regenerative medicine are warranted. Further investigations should explore this promising area of regenerative medicine and should lead to including platelet-rich plasma in treatment guidelines when strong scientific evidence of medical benefit is provided.

To our knowledge, this paper is the first to extensively describe PRP’s mechanisms of action in the dermatology field.
